# Prospective memory: the combined impact of cognitive load and task focality

**DOI:** 10.1007/s00429-023-02658-3

**Published:** 2023-06-25

**Authors:** G. Cantarella, S. Mastroberardino, P. Bisiacchi, E. Macaluso

**Affiliations:** 1 Department of Psychology, University of Bologna, Viale Berti Pichat, 5, 40127 Bologna, Italy; 2 Department of General Psychology, University of Padua, Via Venezia, 8, 35131 Padua, Italy; 3 Department of Human and Educational Sciences, University of Perugia, Perugia, Italy; 4 Padova Neuroscience Center, Padua, Italy; 5 Centre de Recherche en Neurosciences de Lyon CRNL U1028 UMR5292, IMPACT, Université Claude Bernard Lyon 1, CNRS, INSERM, 69500 Bron, France; 6 NeuroImaging Laboratory, Fondazione Santa Lucia IRCCS, Rome, Italy

**Keywords:** Prospective memory, Load, Focality, Monitoring, Detection, Intention

## Abstract

Prospective Memory (PM) entails a set of executive processes primarily associated with the activation of frontal and parietal regions. Both the number of PM-targets to be monitored (i.e. task load) and the relationship between the type of PM-targets and the ongoing (ONG) task (i.e. task focality) can impact executive monitoring and PM performance. In the present imaging study, we manipulated load and focality of an event-based PM task to test the hypothesis that common resources engage in situations requiring high levels of cognitive control: that is, in high-load (i.e. monitor multiple PM-targets) and non-focal conditions (i.e. monitor at the same time letters’ identity and color). We investigated monitoring-related and detection-related processes by assessing behavior and brain activity separately for ONG trials (monitoring) and PM-targets (detection). At the behavioral level, we found a significant interaction between load and focality during detection, with slowest reaction times for focal, high-load PM-targets. The imaging analyses of the detection phase revealed the activation of the left intraparietal sulcus in the high-load conditions. Both in the monitoring and the detection phases, we found overlapping effects of non-focality and low-load in the fusiform gyrus. Our results suggest that under low-load conditions, cognitive control operates via early selection mechanisms in the ventral occipito-temporal cortex. By contrast, high-load conditions entail control at later processing stages within the dorsal parietal cortex. We conclude that load and focality operate via different mechanisms, with the level of task load largely determining how cognitive control selects the most relevant information.

## Introduction

People often refers having poor memory if—for example—they frequently miss appointments or forget to take medications. Our daily routine is full of activities that require remembering to take actions in the future that we generally perform without much effort. This ability to form an intention that needs to be carried out in the future is important for survival, considering that failure to perform such actions may have disastrous consequences.

Prospective Memory (PM) refers to a set of high-order cognitive processes that underlie the realization of intended actions in the future. In a typical PM experiment, participants perform a primary ongoing task (ONG) plus an additional PM task. The PM task comprises performing an action when a specific target is presented (event-based PM) or at a specific time or interval of time (time-based PM). Accordingly, the PM paradigm can be divided into a monitoring phase (when the participant performs the ONG task, while monitoring the occurrence of the PM-target) and a detection phase that includes the detection and response to the PM-target. Behaviorally, the presence of the PM task interferes with the performance on the continuous ONG task, yielding to slower reaction times and/or lower accuracy compared with conditions when the participants perform the very same ONG task but without having to monitor for the PM-target (Brandimonte et al. 2001). These behavioral findings indicate that the performance on the ONG task and the monitoring of the PM-target rely—at least to some extent—on common resources, albeit the subtended mechanisms are not fully understood yet (cf. the so-called “cost debate”: Einstein et al. 2005; Einstein and McDaniel 2010; Smith 2003; Smith et al. 2007; Strickland et al. 2017).

A most influential theory seeking to explain the interplay between ONG and PM performance is the *multiprocess framework* (see McDaniel and Einstein 2000; Einstein and McDaniel 2005). This postulates that different processes subtend complex everyday PM behavior. Specifically, the recruitment of distinct mechanisms (voluntary monitoring vs. spontaneous reflexive-associative processes, see also below) depends on multiple factors, such as the specific characteristics of the PM task, the relative emphasis placed on PM vs. ONG performance, as well as individual differences (Einstein et al. 2005; McDaniel et al. 2015; McDaniel and Einstein 2000).

A key factor known to determine the level of interference between ONG and PM is the ***task load*** associated with the number of potential PM-targets—or target categories—that needs to be monitored to perform the prospective memory task. High-load conditions require the allocation of more resources to strategically monitor the occurrence of the PM-targets (Einstein et al. 1992; 2005). For example, if the load of the PM task is increased by asking participants to monitor for two different PM-targets, performance of the ONG task decreases (West and Bowry 2005; West et al. 2006). This is consistent with the notion that common cognitive resources underlie the monitoring of the PM-targets and the execution of the ONG task (see also Barban et al. 2014).

Another factor that plays a role in determining the interaction between ONG and PM concerns the relationship between the two tasks. This is referred to as ***task focality*** (focal vs. non-focal) and concerns whether the ONG task and the PM-target require judging the same vs. different dimensions of the stimuli (e.g. Einstein et al. 2005; Einstein and McDaniel, 2005; Cohen et al. 2017; Hicks et al. 2017; Scullin et al. 2010, Cona et al. 2014). For example, if the ONG task requires performing a lexical decision, a focal PM-target may involve the detection of a specific word, while a non-focal target could entail the detection of a specific syllable (e.g. Barban et al. 2020). Consistent with the *multiprocess framework*, focal and non-focal targets can yield to different levels of interference between ONG and PM, typically with more interference for non-focal than focal conditions (Scullin et al. 2010).

At the brain level, the cost of performing a PM task can be studied by comparing activity when the participants perform the ONG task alone versus when they also have to monitor for the PM-targets. Such comparison revealed activation of a broad network of brain areas comprising high-level associative regions in the frontal and parietal cortex, as well occipito-temporal regions, including the lingual, fusiform, and parahippocampal gyri (Okuda et al. 1998; Gonneaud et al. 2014). In the frontal cortex, a key region is the anterior prefrontal cortex (aPFC, Broadmann’s area 10), which plays a crucial role in maintaining delayed intentions (e.g. Burgess et al. 2001; 2003; den Ouden et al. 2005; Gilbert et al. 2009; Momennejad and Haynes 2012; Okuda et al. 1998; Burgess et al. 2011) and is connected with attention controlling regions in the frontoparietal cortex. In the *multiprocess framework*, these frontoparietal regions exert goal-directed, top–down control to maintain the PM intention and to monitor for the occurrence of the relevant PM-targets (Cona et al. 2015). By contrast, the detection of the PM-targets and the execution of the corresponding action has been related primarily to the engagement of the ventral parietal cortex (BA 40). For example, using transcranial magnetic stimulation (TMS), Bisiacchi and colleagues (2011) showed that stimulation of the left inferior parietal lobule interfered selectively with the retrieval of intentions, slowing down the execution of the intended action, whereas the ONG performance in the monitoring phase was unaffected.

In line with this dorsal/ventral dissociation, Cona and colleagues (2015) proposed the "Attention to Delayed Intentions model" (*A-to-DI* model, Cona et al. 2015). This postulates that dorsal fronto-parietal regions mediate top–down, voluntary mechanisms during the monitoring phase, while ventral fronto-parietal regions would instead engage in a bottom–up, automatic manner during the detection of the PM-targets. Nonetheless, as implied in the *multiprocess framework*, the relative contribution of voluntary vs. automatic processing, and thus the involvement of separate brain circuits, may vary as a function of task constraints both in the monitoring and the detection phases.

Several imaging studies showed that task ***focality*** can affect areas activated during the monitoring phase (McDaniel et al. 2013; Beck et al. 2014; Rusted et al. 2011; Barban et al. 2020). Barban and colleagues (2020) reported a dissociation within the aPFC, with activation of the lateral aPFC for the non-focal conditions and of the medial aPFC for the focal conditions. Furthermore, the same study also found that the non-focal conditions activated the intraparietal sulcus (IPS), the middle frontal gyrus (MFG), and the supplementary motor cortex (SMA), while the focal conditions were associated with the activation of ventro-medial prefrontal cortex (vmPFC). The ***load*** of the PM task has also been found to affect activity associated with the ONG trials in the monitoring phase. An EEG study (Hering et al. 2020) showed that a fronto-central sustained negativity during ONG trials became less negative when increasing the number of prospective intentions. This effect of PM-load on brain activity during the performance of the ONG task was related to the augmented attentional and mnestic processes required to maintain the increasing number of prospective intentions (see also Kuhlmann and Rummel 2014; Cona et al. 2014).

In the detection phase (PM-targets), task ***focality*** has been found to modulate activity of the aPFC and the middle temporal cortex. Activity in the lateral aPFC was found to increase in non-focal compared to focal tasks (McDaniel et al. 2013) which, according to the *gateway hypothesis* (Burgess et al. 2007), has been interpreted as the correlate of a processing bias toward internal representations. Conversely, focal tasks have been associated with the modulation of middle temporal regions (McDaniel et al. 2013; Reynolds et al. 2009). In particular, McDaniel and colleagues (2013) suggested that the transient activation of the middle temporal gyrus mediates the suspension of the processing of the ongoing stimuli, shifting the attentional focus toward the focal PM-targets. To investigate the impact of ***load*** on detection activity, Barban and colleagues (2014) compared blocks requiring the monitoring of four PM-targets (four target letters; high memory load) vs. one target (one letter only; low memory load). During target detection, the high PM-load was associated with activation of the left posterior frontal lobe, the left middle temporal gyrus, the precuneus, and the inferior parietal lobe bilaterally; while the low-load condition activated a network of medial and ventral regions, also including the occipital pole. High PM-load has also been associated with the dorsal parietal cortex, specifically in the left hemisphere. Using TMS, Cona and colleagues (Cona et al. 2017) compared TMS stimulation of the left vs. right dorsal parietal cortex in conditions involving either "retrospective load" (i.e. a condition meant to engage attention toward internal representations) or "monitoring load" (i.e. a condition targeting attention to external stimuli). The results showed that TMS affected PM-target detection in both load conditions, but only when applied over the left hemisphere.

**Fig. 1:**
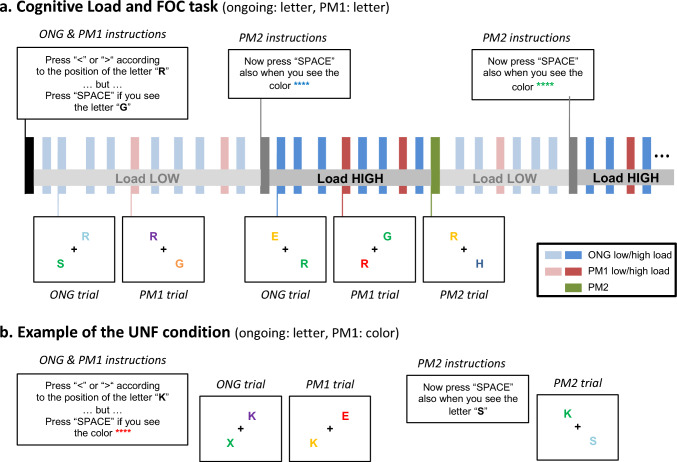
Task, stimuli and conditions. **A** Example of a sequence of trials for the focal condition (FOC), showing the load changes (HIGH/LOW). At the beginning of the fMRI-run, participants received the instructions concerning the ongoing task (ONG), plus one prospective memory target (PM1). In the example, the ONG task required pressing left or right button with the right hand (“ < ” or “ > ”) according to the position of a specific letter (“R"), while concurrently monitoring for the appearance of the PM1-target (“G”) that required pressing a different button with the left hand (labeled "SPACE"). At unpredictable times during the execution of the ONG/PM1 tasks, the PM2 instructions appeared on the screen. This indicated to the participants that, together with ONG and PM1, they also had to monitor for another PM-target (PM2) that also required pressing the left-hand button ("SPACE"). The PM2 always involved judging a different dimension than PM1, thus color in this example ("blue" for the first PM2, "green" for the second PM2). The presentation of the PM2 instruction implied an increase of Load (low to high), while the correct detection of the PM2 target implied a decrease of load (high to low). **B** Example of trials for the non-focal condition (UNF) that was presented in separate fMRI-runs. The ONG task was the same as in the focal condition (discriminate the position of a target letter, here "K"), but now the PM1-target required monitoring a different dimension of the stimuli, namely color ("red", in the example). As in the focal condition, the PM2 instructions appeared on the screen at irregular intervals, requiring the participants to also detect a second type of PM-target (PM2). Note that because PM1 and PM2-targets always involved judging different dimensions, now the PM2-targets comprised the detection a specific letter ("S", in the example). Again, the presentation of the PM2 instructions and the correct detection of the PM2-targets served to switch the level of load (high/low)

In sum, previous work pointed to a dissociation between monitoring-related processes in the dorsal fronto-parietal cortex vs. detection-related processes in the ventral parietal cortex. Nonetheless, such straightforward dissociation cannot account for conditions when competition between ONG and PM arises from multiple sources (e.g. *Load* and *Focality*). In the present study, we sought to characterize how the co-occurrence of *Load* and *Focality*, both impacting on executive demands, affects behavior and brain activity associated with monitoring and detection. During fMRI, the participants were asked to press a left or right button according to the position of a specific letter (*ONG task*), while remembering to press a different key upon the appearance of different specific letter (i.e. the *focal PM1-target*) or a letter of a specific color (*non-focal PM1-target*). Focal and non-focal conditions were presented in separate sessions/fMRI-runs. Within each run, participants were presented with the additional instruction to detect a second type of PM-target (*PM2 target,* either *focal* or *non-focal* with respect to the ONG task), at irregular intervals during task performance, thus increasing PM task load. These experimental manipulations allowed us to investigate the effects of *Focality* and *Load* on the processing of the ONG trials, and the interaction between *Focality* and *Load* during the detection of the PM1-targets (see “Methods” section for a detailed description of all the different trial types, cf. also Fig. 1).

Our main prediction was that if the effects of *Load* and *Focality* rely on shared cognitive resources, the two factors should activate overlapping brain regions and—possibly—reveal a statistical interaction during the detection of non-focal PM-targets in the high-load condition, when shared resource would be greatly stimulated. Common executive processes may involve regions of the dorsal parietal cortex that have been previously associated both with high-load and non-focality (cf. above), as well as anterior prefrontal regions that have been associated with the control of resources between internal/intentional and external/sensory sources of information (Burgess et al. 2003; McDaniel et al. 2013).

## Materials and methods

### Participants

Eighteen right-handed volunteers were recruited for the study. All participants were neurologically intact, free of psychotropic or vasoactive medication, and with no history of psychiatric or neurological disease. They had normal or corrected-to-normal vision (i.e. contact lenses). Before scanning, all participants underwent a 20 min training session to familiarize with the task. In the training session, a feedback was provided for each response. All participants achieved 80% correct responses during the training phase. Nonetheless, during fMRI scanning, three participants failed to respond to all the trials belonging to one of the PM conditions (see below) and were excluded from the analyses. The final analyses included 15 participants (8 males; mean age = 24.5 ± 3.5). The study was approved by the independent Ethics Committee of the IRCCS Fondazione Santa Lucia (Scientific Institute for Research Hospitalization and Health Care) and all participants gave written informed consent before the beginning of the experiment.

### Experimental paradigm

During the experiment, participants were asked to press a left or right button with the right hand according to the position of a specific letter (*ongoing task* **[ONG]**: e.g. letter “R", see Fig. 1A). Concurrently, they were also asked to monitor the appearance of additional targets (prospective memory targets: PM1 and PM2) that required withholding the response to the ONG task and pressing a different button with the left hand instead. In different fMRI-runs, the PM1 task included detecting targets of the same category as the ONG task (*focal task condition* **[FOC]**: ONG-letter/PM1-letter, see Fig. 1A) or targets of a different category (*non-focal task condition* **[UNF]**: ONG-letter/PM1-color, Fig. 1B). The category of the PM2-target was always opposite to that of the current PM1 (i.e. PM1-letter/PM2-color, or PM1-color/PM2-letter; see Fig. 1).

At the beginning of each fMRI-run, participants received instructions about the ONG task, as well as instructions about the PM1-target that they had to detect throughout the entire fMRI-run. Participants were also informed that during the fMRI-run, after a variable number of trials, an additional instruction to monitor and detect a second type of PM-target (PM2-target) would appear and that as soon as they detected the PM2-target they had to stop monitoring for it. Accordingly, throughout the run, participants performed the ONG task and monitored either one PM-target (*low-load condition* **[LOW]**: PM1 only) or they monitored two different PM-targets (*high-load condition* **[HIGH]**: PM1 and PM2). It is important to note that all our analyses considered that *Load* changed from HIGH to LOW only when the participant correctly detected the PM2 target, see also Fig. 1A for a graphic illustration of the change *Load* over a sequence of trials.

The combination of these different manipulations led to four types of PM1 trials that allowed us to address the issue about the interplay between *Load* and *Focality* during PM detection (HIGH/LOW x FOC/UNF). The ONG trials were also presented under HIGH/LOW load and either in FOC/UNF conditions. Nonetheless, it should be noted that in the HIGH load condition, participants had to monitor also the PM2-targets that always involved a different category than PM1 (see above). Therefore, ONG trials in HIGH load implied monitoring two different PM categories (i.e. letters and colors) and were therefore all considered as non-focal (UNF). Because of this, the analyses of the ONG trials could address the effects of *Load* and *Focality*, but not the interaction between the two factors (see also below for further details).

### Stimuli and task

Stimulus presentation was controlled with MATLAB 7.1 (The MathWorks Inc., Natick, MA), using Cogent2000 Toolbox (Wellcome laboratory of Neurobiology, University College London). The visual stimuli were presented on a black background using a rear projection system (total display size = 20 degrees of visual angle, 1024 × 768 screen resolution, and 60 Hz refresh rate).

Each trial included the presentation of two colored letters on the left and right side, upper or lower position, of the central fixation (e.g. see Fig. 1). Stimuli were presented for 1000 ms with an inter-trial interval of 500 ms. The display always contained the to-be-judged target stimulus for the ONG task. The participants responded with the right hand, pressing the left or right button of an MR-compatible button box to indicate whether the ONG target letter was on the left/right side of the screen. On PM1 trials (6% of the trials), in addition to the ONG stimulus, the display included also the PM1-target that was cued at the beginning of the fMRI-run. The participants had to withhold the right-hand response and press a third button with the index finger of the left hand to indicate that they had detected the PM1-target.

At irregular intervals (range 7–36 trials), a new instruction display was presented (duration 3 s), indicating that a second PM-target required monitoring and responding with the left-hand index finger (PM2-target, 5% of the trials). After the PM2 instruction, the trial presentation resumed, now with the participants monitoring two different PM-targets while performing the ONG task (high-load condition). To avoid that participants could anticipate whether the next PM-target would be a PM1 or a PM2, the PM1/PM2 presentation between two PM2 instructions was counterbalanced in six possible sequences: just the PM2 without any PM1, one PM1 before the PM2; two PM1 before PM2; one PM1 after the PM2; two PM1 after the PM2, one PM1 before and one PM1 after the PM2. Critically, when the PM1 was presented before the PM2, this was a high-load PM1; while when it was presented after a (correctly detected) PM2, the PM1-target was low-load, cf. Fig. 1A. The number of ONG trials between PM-targets ranged between 3 and 8. The high/low load of the ONG trials also depended on whether these were presented before/after the (correctly detected) PM2-targets; see Fig. 1A.

Each run included 456 trials: 405 ONG trials (180 LOW load, and 225 HIGH load), 27 PM1-targets (15 LOW load and 12 HIGH load), and 24 PM2-targets. Each participant underwent 4-fMRI-runs: two runs with PM1-letter (FOC condition) and two runs with PM1-color (UNF condition).

### Behavioral data

For each participant, mean Reaction Times (*RTs*) and accuracy (*ACC*) were computed using MATLAB 7.1 (*The MathWorks Inc., Natick, MA*). Statistical analyses were performed with SPSS v.21.

The main analysis targeted the PM1 trials, for which we could investigate the interaction between *Load* and *Focality*, considering four trial types: PM1_Hfoc, PM1_Lfoc, PM1_Hunf, and PM1_Lunf. The data analysis comprised two repeated-measures 2 × 2 ANOVAs with the factors *Load* (HIGH/LOW) and *Focality* (FOC/UNF), separately for RTs and ACC. The emerging significant interactions among the factors were followed by post hoc analyses with Bonferroni’s correction to reduce the Type I error rate.

For the PM2 trials, paired-samples T tests compared PM2_Hfoc and PM2_Hunf trials, again considering separately RTs and ACC. This allowed us to assess the behavioral effect of *Focality* also on the detection of the PM2-targets.

Finally, we examined the effects of *Load* and *Focality* on the ONG trials. For this, we computed paired-samples T tests: ONG_Lfoc versus ONG_Lunf (effect of *Focality*) and ONG_Lunf versus ONG_Hunf (effect of *Load*), considering RTs and ACC as the dependent variables.

### fMRI acquisition and pre-processing

Images were acquired with a Siemens Allegra (Siemens Medical Systems, Erlangen, Germany) 3 T scanner. Functional imaging data were acquired using echo-planar imaging (32 contiguous transverse slices covering the entire cerebral cortex with TR = 2.08 s, echo time = 30 ms, flip angle = 70°, 64 × 64 matrix, voxel size = 3 × 3 mm in-plane, slice thickness = 2.5 mm; 50% distance factor). Head movement was minimized by mild restraint and cushioning. Each participant underwent four fMRI-runs, each comprising 374 volumes.

Data were preprocessed and analyzed with SPM12 (*Wellcome Trust Centre for Neuroimaging, London, UK*). The first four scans of each fMRI-run were discarded to allow the signal to stabilize. The remaining scans were corrected for motion between slices and for slice-timing acquisition differences using the middle slice as a reference. Images were normalized to the Montreal Neurological Institute (MNI) standard space. Normalization parameters were estimated using the mean EPI and then applied to all functional images. The images were spatially smoothed with an isotropic Gaussian kernel of 8 mm FWHM (full-width half-maximum).

### fMRI analyses

Statistical inference was based on a random-effects approach (Penny and Holmes 2007), which comprised two steps: first-level analyses estimating contrasts of interest for each subject, followed by second-level analyses for statistical inference at the group level (with non-sphericity correction, Friston et al. 2002).

For each participant, the first-level general linear model (GLM) included 12 conditions reflecting activity for PM1, ONG, PM2, and PM2 instructions. The PM1 trials were modeled using 5 predictors: 4 arising from the combination of load (HIGH/LOW) and focality (FOC/UNF) and including correct trials only (PM1_Hfoc, PM1_Hunf, PM1_Lfoc, and PM1_Lunf) and 1 including all incorrect PM1 trials (wPM1). The ONG trials were modeled with 4 predictors: 3 correct conditions (ONG_Lfoc, ONG_Lunf and ONG_Hunf; considering that all ONG trials in the high-load condition involved monitoring of PM1/PM2-targets of a different category: letter and color, see also Sect.  “Experimental paradigm”), plus the incorrect trials (wONG). The PM2 trials were modeled with 3 predictors, comprising correctly detected targets as a function of focality (PM2_Hfoc, PM2_Hunf), plus the incorrect trials (wPM2). Note that, by definition, the PM2 involved detection under the high-load condition; see Fig. 1. A separate predictor modeled the PM2 instructions.

Each trial was modeled as an event time-locked to the onset of the visual display, with a zero duration for ONG and PM trials, and 3000 ms for the PM2 instructions. All trials were convolved with the SPM12 hemodynamic response function. It should be noticed that because of the relatively short stimulus onset asynchrony (1500 ms, cf. above), the GLM predictors included overlapping hemodynamic responses between the different conditions. However, the estimation of the parameters estimates of the GLM takes these dependencies (co-variation) into account, ensuring that when conditions are compared, the resulting activations refer specifically to variance that is uniquely associated with one or the other condition (i.e. any shared variance fitted by multiple predictors is factored out). The six parameters of head movements resulting from the rigid-body realignment were included as covariates of no interest. The time series at each voxel were high-pass filtered at 120 s and pre-whitened by means of autoregressive model AR(1).

For each participant, we performed 3 sets of contrasts that were subsequently entered in 3 separate II-level analyses for statistical inference at the group level. A preliminary analysis directly compared monitoring and detection activity and tested for the effect of accurate task performance. For this, we computed 6 contrasts at the first level. These averaged separately all the correct ONG conditions, correct PM1, correct PM2, and the three corresponding error trials (wONG, wPM1, and wPM2). The second analysis tested for the effects of *Load* and *Focality* during the ONG task (monitoring). The first-level contrasts averaged the parameters estimates for the three relevant conditions across fMRI-runs, considering correct trials only (ONG_Lfoc, ONG_Lunf, and ONG_Hunf). Finally, we addressed our main hypothesis with the third analysis that tested for the effects of *Load* and *Focality*, and their interactions, during the detection of the PM-targets. For this we considered the 6 conditions associated with correct PM trials (PM1_Hfoc, PM1_Hunf, PM1_Lfoc, PM1_Lunf, PM2_Hfoc, and PM2_Hunf). The first-level contrasts averaged the parameters estimates across the two fMRI-runs that included the relevant conditions.

The three sets of contrasts were entered in 3 separate repeated-measures ANOVAs. With the first ANOVA, we highlighted the effect of monitoring by comparing "correct ONG vs. correct PM" trials, and the effect of detection comparing "correct PM vs. correct ONG". Note that these two comparisons basically comprise the two tails of the same t test that in SPM are assessed using two separate group-level contrasts, revealing the voxels that activate for either "ONG > PM" or "PM > ONG". To ensure that the reported activations are specific for correct performance of the monitoring and detection processes, the contrasts were masked inclusively (p-unc = 0.005) with the corresponding effects of accurate task performance. That is, for the monitoring contrast (ONG vs. PM), the mask was "correct ONG > wrong ONG"; while for the detection contrast (PM vs. ONG), the mask was "correct PM > wrong PM". In these analyses, all PM contrasts averaged the PM1 and PM2 conditions, albeit these are plotted separately in Fig. 3a with the aim of providing some additional information about the contribution of PM1 and PM2 to the reported effects (see below for tests targeting PM1 and PM2, separately).

The second ANOVA included the 3 ONG conditions. As noted above, the ONG trials in high-load required monitoring simultaneously two PM-targets and were all modeled as a single ONG_Hunf condition in the first-level models. Because of this, the effect of *Focality* considered only the low-load conditions ([ONG_Lunf > ONG_Lfoc], and viceversa) and the effect of *Load* considered only non-focal conditions ([ONG_Hunf > ONG_Lunf], and vice versa).

The third ANOVA addressed our main hypothesis concerning *Load* and *Focality* during PM detection. The analysis included 6 conditions: PM1_Hfoc, PM1_Hunf, PM1_Lfoc, PM1_Lunf, PM2_Hfoc, and PM2_Hunf. To test for the combined effects of high-load and non-focal task during PM1 detection, we considered the interaction contrast: [PM1_Hunf-PM1_Hfoc > PM1_Lunf-PM1_Lfoc]. Within the same model, we assessed the effect of *Load* by comparing all trials with PM-targets presented under "High vs. Low" load (e.g. for HIGH load: [PM1_Hfoc + PM1_Hunf + PM2_Hfoc + PM2_Hunf > 2*(PM1_Lfoc + PM1_Lunf)]. Analogously, for the effect of *Focality,* we compared all "Focal vs. Non-focal" PM trials (e.g. for the non-focal condition: [PM1_Hunf + PM1_Lunf + PM2_Hunf > PM1_Hfoc + PM1_Lfoc + PM2_Hfoc]. Finally, we directly compared the PM1 and the PM2 conditions considering the high-load PM1 trials ([PM1_Hfoc + PM1_Hunf > PM2_Hfoc + PM2_Hunf]).

The statistical thresholds were set to p-FWE-corr. = 0.05 at the cluster-level (cluster-size estimated at p-unc. = 0.005), corrected for multiple comparison at the whole-brain level. The cluster-level correction procedure takes into account both the "strength/height" of activation peak and the spatial extent of the activation (i.e. the cluster-size), making this test sensitive both to sharp localized signals and to spatially extended signals (Poline et al. 1997). In addition, because we had expectations about the role of the dorsal parietal cortex during the detection of high-load PM-targets and the possible interaction between *Load* and *Focality* in the same region (see “Introduction”), for these two contrasts, we also employed a more targeted Region of Interest (ROI) approach. We used the AnatomyToolbox (Eickhoff et al. 2005) to construct an ROI comprising the left IPS (areas hIPS1, hIPS2, and hIPS3 in the AnatomyToolbox). Here, we focused specifically on the left hemisphere based on the results of Cona and colleagues (Cona et al. 2017), who reported that only TMS stimulation of the left dorsal parietal cortex affected the processing of PM-targets under high-load conditions. The ROI data were extracted and analyzed with the MarsBaR toolbox (Brett et al. 2002) that averages activity of all the voxels in the ROI.

## Results

### Behavioral results

**Fig. 2:**
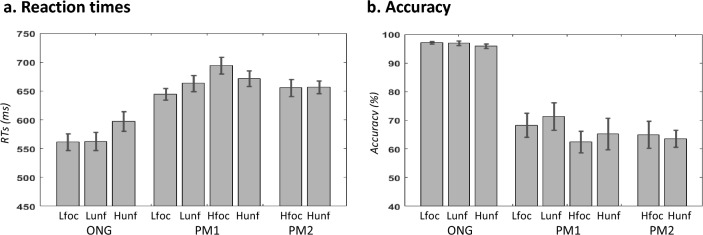
Behavioral performance. Reaction times (**a**) and accuracy **b** for each experimental condition (mean, + /– std). *ONG*/*PM* ongoing task/prospective memory targets, *L/H* low-/high-load, *foc/unf* focal/non-focal

Overall, the discrimination accuracy of the ONG task was high (> 95%), while the detection accuracy of the PM-targets was relatively low (< 70%, see Fig. 2). Most of the ONG errors were either incorrect left/right discriminations or false alarms (button-presses associated with the PM-targets). The vast majority of the errors in the PM task comprised target-omissions, with the participants responding to the ONG task (82% of the PM errors). Below, we present the results of the behavioral analyses that assessed the effects of *Load* and *Focality*, separately for the PM and ONG tasks.

#### Prospective memory trials

To investigate the effects of *Load* and *Focality* on detection, we examined the responses to the PM-targets. First, two separate repeated-measures ANOVAs considered the RTs and accuracy for the 4 types of PM1-targets (see bars 4–7 in Fig. 2). The ANOVA on RTs revealed a main effect of *Load* (F (1, 14) = 27.69, *p* < 0.001, η^2^_p_ = 0.664) and a significant *Load x Focality* interaction (F (1, 14) = 6.69, *p* = 0.022, η^2^_p_ = 0.323). The main effect of *Focality* was not significant (F (1, 14) = 0.42, *p *= 0.841, η^2^_p_ = 0.003). Post hoc tests revealed a significant effect of the *Load* only for the focal trials (t (14) = 4.4; *p* < 0.001) that explains this interaction, see "bar 5 vs. bar 4" (effect of HIGH load in FOC) as compared with "bar 7 vs. bar 6" in Fig. 2a.

The corresponding ANOVA on accuracy confirmed the main effect of *Load* (F (1, 14) = 15.58, *p* = 0.001, η^2^_p_ = 0.527), with lower accuracy for the high-load compared with low-load condition, while the *Load x Focality* interaction was not significant (F (1, 14) = 0.01, *p* = 0.945, η^2^_p_ = 0.000). Again, the main effect of *Focality* was not significant (F (1, 14) = 0.36, *p* = 0.559, η^2^_p_ = 0.025).

Finally, we tested the effect of *Focality* considering the detection of the PM2-targets (PM2_Hunf vs. PM2_Hfoc). Both the *t* tests on the RTs and on accuracy were not significant [RTs: t (14) = 0.11, *p* = 0.918; accuracy: t (14) = 0.37, *p* = 0.720].

#### Ongoing trials

Two separate analyses considered the effects of *Load* and *Focality* during the ongoing task, aiming to understand how these factors impacted on the monitoring of prospective memory intentions. To investigate the effect of *Load*, we considered only the non-focal conditions and directly compared ONG_Hunf vs. ONG_Lunf trials. A paired-samples T test showed that the RTs were significantly slower in the HIGH load trials compared to the LOW load trials (t(14) = 6.82, *p* < 0.001, compare bar 3 vs. bar 2, in Fig. 2a). The corresponding analysis on accuracy indicated only a marginal effect of load (t(14) = 1.61, *p* = 0.131; Fig. 2b, but note that overall performance was almost at ceiling,  > 95%). The tests for the effect of *Focality* considered only the LOW load trials (ONG_Lunf vs. ONG_Lfoc). Neither the RTs nor the accuracy tests reached statistical significance (RTs: t(14) = 0.23, *p* = 0.824; accuracy: t(14) = 0.16, *p* = 0.878).

### fMRI results

The main aim of the study was to identify the impact of *Load* and *Focality* on the brain circuits involved in different phases of prospective memory intentions: detection and monitoring. For the detection phase, we considered brain activity associated with processing of the PM-targets, while for the influence of these factors on monitoring, we examined activity during the ONG trials. As a preliminary analysis, we directly compared PM and ONG trials to reveal networks that activated differentially during monitoring and detection.

#### Monitoring and detection networks

We directly compared ONG trials vs. PM trials to identify brain networks involved in monitoring vs. detection, irrespective of *Load* and *Focality*. Beside considering only correct trials, we also masked the relevant contrasts with the corresponding effects of performance accuracy (correct vs. wrong trials), thus ensuring that we identified areas specifically engaged during correct performance.

**Fig. 3:**
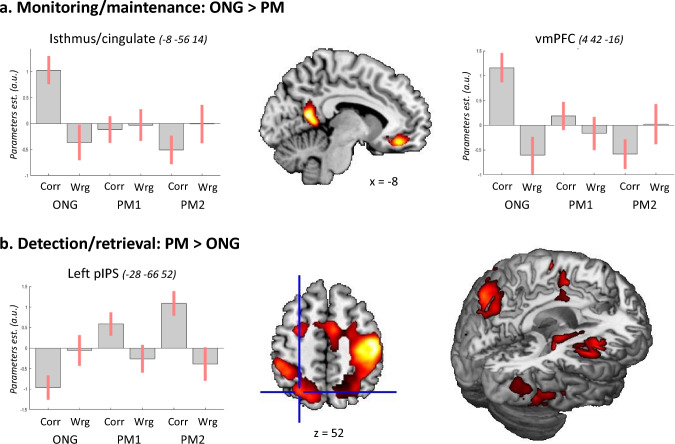
Overall effects of monitoring vs. detection. **a** Effect of monitoring (ONG > PM) inclusively masked with the effect of correct performance of the ongoing task (correct > wrong). The results highlighted two significant clusters: one in the medial parietal cortex (isthmus/cingulate) and one in the ventro-medial prefrontal cortex (vmPFC). The signal plots show that activity in these regions was selective for ONG trials with correct responses (cf. bar 1, in both plots). **b** Effect of detection (PM > ONG), masked with the effect of correct PM detection. This comparison highlighted an extensive network of brain areas, including the dorsal fronto-parietal cortex (see central panel), occipital areas, insula, putamen, and SMA (visible in the 3D-render, rightmost panel); see also Table 1. The signal plot shows the pattern of activation in the left posterior intraparietal sulcus (pIPS), where the strongest response was found for the correct detection of PM1 and PM2-targets (bars 3 and 5). The signal plots show the parameters estimates of the general linear model ± 95% CI. Note that because of the repeated measure, the parameter estimates sum to zero and negative values should not be interpreted as de-activations. Activation clusters are significant at *p*-FWE-corr. = 0.05 (cluster level) and are displayed at a voxel-level *p*-unc. = 0.005. Corr/Wrg: correct/wrong

The contrast "ONG > PM" (pooling PM1 and PM2 and masked with the contrast "correct ONG > wrong ONG") revealed a cluster of activation in the ventro-medial prefrontal cortex (gyrus rectus) and a second cluster in the medial parietal cortex (see Fig. 3a and Table 1). The anterior cluster comprised the areas BA10 and BA11, and the posterior cluster comprised the isthmus/posterior cingulate cortex. The opposite contrast revealed an extensive pattern of activation associated with the detection of the PM-targets (PM > ONG, Fig. 3b). The activated areas comprised regions of the dorsal fronto-parietal network, including the intraparietal sulcus and dorsal premotor cortex, the lateral occipital cortex, the right insula, the caudate and the supplementary motor area (SMA), plus the right sensory–motor cortex and the left cerebellum; see also Table 1. The occipital activation extended from the inferior occipital gyrus, anteriorly to the fusiform gyrus. The activation of the right sensory–motor cortex and the left cerebellum is consistent with the left-hand responses that characterized the correct detection of the PM-targets.

**Table 1:** Peak activations for the contrasts ONG > PM and PM > ONG at *p* < 0.05 FWE-corrected at the cluster level (cluster defining threshold, *p*-unc. = 0.005)

	Region	Size	MNI cords (mm)			*t*-stat	*p*_*FWEcl*_
			*x*	*y*	*z*		
Monitoring: ONG > PM							
1	Isthmus/cingulate	987	− 8	− 56	14	6.26	0.001
2	Gyrus rectus	689	4	42	− 16	5.80	0.009
Detection: PM > ONG							
1	L cerebellum	3359	− 24	− 50	− 24	13.53	< 0.001
	L IOG		− 44	− 68	− 12	7.71	
	L FusG		− 46	− 56	− 12	6.40	
2	R sensory–motor	11960	44	− 16	56	13.26	< 0.001
	R aIPS		40	− 32	62	11.83	
	R dPM		32	− 10	64	10.19	
	SMA		6	2	46	8.22	
	R POp		50	− 18	18	8.22	
	R putamen		14	− 18	8	7.50	
	R pIPS		32	− 64	46	6.88	
	R insula		40	0	10	6.70	
3	L aIPS	2966	− 46	− 34	42	9.81	< 0.001
	L pIPS		− 28	− 66	52	7.62	
4	L dPM	573	− 24	0	62	8.33	0.021
5	R IOG	1550	36	− 66	− 15	6.07	< 0.001
	R FusG		44	− 52	− 14	5.91	

The contrasts are masked inclusively (p-unc. = 0.005) with the corresponding effects of performance accuracy (e.g. "correct ONG > incorrect ONG", for "ONG > PM"). The table reports the MNI coordinates (x y z), *t*-statistic (*t* stat), and p-statistic (p_FWEcl_). Cluster sizes are in number of voxels (Size). *IOG/FusG* inferior occipital gyrus/fusiform gyrus, *a/pIPS* anterior/posterior intraparietal sulcus, *dPM* dorsal premotor cortex, *SMA* supplementary motor area, *POp* parietal operculum, *L/R* left/right hemisphere

#### Detection: load and focality in the PM trials

**Fig. 4:**
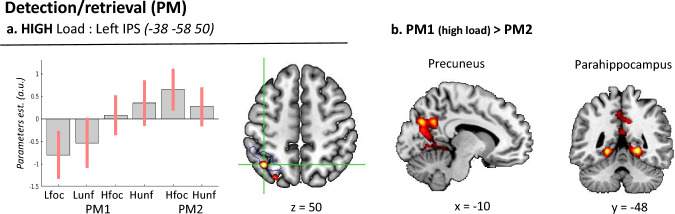
Effects of Load and Focality during detection. **a** The left intraparietal sulcus showed an effect of HIGH load across PM1 and PM2 (compare bars 3–6 vs. bars 1–2). The signal plot refers to the peak voxel located in the left IPS-ROI (see Methods), which here is shown in a blue contour. **b** The direct comparison between PM1 (high-load) and PM2 revealed activation of the precuneus, the parahippocampus, plus the right hippocampus and right insula (not shown here). Activations are displayed at a voxel-level p-unc. = 0.005. The signal plots show mean-adjusted parameters estimates + /– 95% CI

We made use of a focused ROI analysis to assess the predicted effect of *Load* in the left IPS (see “Introduction” and “Methods” section). The "high > low" *Load* comparison was significant (*T* value = 2.37, *p* < 0.011), confirming the engagement of the left IPS in conditions entailing high levels of prospective memory load. Beside the expected engagement of the left IPS, we asked whether the high-load PM trials activated any other area of the brain. The corresponding whole-brain analysis did not reveal any significant effect, after correction for multiple comparisons. Figure 4a shows the pattern of activity of the peak voxel within the left IPS-ROI (cf. blue contour), highlighting larger activation when the participants detected PM-targets in the four high-load conditions compared with the two low-load conditions (cf. bars 3–6 vs. bars 1–2, in the signal plot).

The main effect of *Focality* during PM detection was tested at the whole-brain level and revealed a significant activation cluster in the medial occipital cortex for the non-focal conditions (*x*, *y*, *z* = − 6 − 78 18, *T* = 4.66, *p*-FWE-corr < 0.001).

Next, we turned to the interaction between *Load* and *Focality* expecting to highlight brain areas deploying any common cognitive resource for PM detection under the non-focal and high-load condition. First, we tested the interaction in the anatomically defined left IPS-ROI, which did not reveal any significant effect (*T* value = 0.86, *p* > 0.19). We then run the corresponding test at the whole-brain level, but no cluster survived the correction for multiple comparisons. Nonetheless, when we examined the effect of *Focality* separately under high and low *Load* conditions, we found a significant effect of UNF vs. FOC specifically under the LOW load condition. The cluster was located in the left fusiform gyrus (*x*, *y*, *z* = − 32 − 54 − 16, *T* = 4.16, *p*-FWE-corr. = 0.009), where we also found related effects of focality under low-load during the monitoring phase; see Sect.  “Monitoring: load and focality in the ONG trials”.

In the same model, we directly compared PM1 and PM2, considering specifically the PM1 high-load trials (because PM2-targets were always presented at high-load). The contrast "PM1(high) > PM2" highlighted activation of the precuneus and the parahippocampus bilaterally (see Fig. 4b), with a second cluster comprising the right hippocampus and the right insula (see Table 2). The reverse comparison, "PM2 > PM1(high)", did not reveal any significant activation.

**Table 2:** Peak activations for the detection of (high-load) PM1 vs PM2-targets at *p* < 0.05 FWE-corrected at the cluster level (cluster defining threshold, *p*-unc. = 0.005) with MNI coordinates (x y z), *t*-statistic (*t*-stat), and *p*-statistic (p_FWEcl_)

	Region	Size	MNI cords (mm)			*t*-stat	*p*_*FWEcl*_
			*X*	*Y*	*z*		
Detection: PM1 high vs PM2							
1	R parahippocampal gyrus	3487	20	− 48	− 4	4.69	< 0.001
	L parahippocampal gyrus		− 20	− 48	− 4	4.59	
	Precuneus		− 10	− 74	32	4.27	
2	R hippocampus	479	20	− 18	− 8	3.84	0.018
	R insula		32	− 14	4	3.74	

Cluster sizes are in number of voxels (Size). *L/R* left/right hemisphere

#### Monitoring: load and focality in the ONG trials

As for the analysis of the behavioral data, we assessed the effect of *Load* using the non-focal ONG trials (ONG_Hunf vs. ONG_Lunf) and the effect of *Focality* using the low-load ONG trials (ONG_Lunf vs. ONG_Lfoc).

The effect of high-load (ONG_Hunf > ONG_Lunf) did not reveal any significant activation, while the effect of low-load (ONG_Lunf > ONG_Hunf) highlighted an extensive pattern of activation including ventral occipital–temporal regions (lingual and fusiform gyrus), subcortical areas (putamen and head of the caudate), the insula bilaterally, the left middle temporal gyrus, plus the cingulate cortex; see Table 3.

**Table 3:** Peak activations for low- vs high-load (non-focal) ONG trials at *p* < 0.05 FWE-corrected at the cluster level (cluster defining threshold, *p*-unc. = 0.005) with MNI coordinates (*x* *y* *z*), *t*-statistic (t-stat), and p-statistic (p_FWEcl_)

	Region	Size	MNI cords (mm)			*t*-stat	*p*_*FWEcl*_
			*x*	*y*	*Z*		
Monitoring: low vs. high load (non-focal ONG)							
1	L insula	3057	− 36	− 10	14	6.41	< 0.001
	L MTG		− 48	− 6	− 24	6.08	
	L putamen		− 26	4	10	4.55	
	L caudate		− 12	10	12	4.22	
2	R LingG	7012	14	− 76	− 8	6.38	< 0.001
	R FusG		28	− 52	− 14	6.30	
	L FusG		− 28	− 58	− 10	6.06	
	R caudate		16	16	8	5.04	
	L LingG		− 8	− 80	− 6	5.00	
	R putamen		20	14	2	4.63	
	R insula		32	− 12	16	4.61	
3	L cingulate gyrus	2741	− 12	− 34	54	6.14	< 0.001
	R cingulate gyrus		14	− 30	48	5.52	

Cluster sizes are in number of voxels (Size). *MTG* middle temporal gyrus, *LingG* lingual gyrus, *FusG* fusiform gyrus, L/R left/right hemisphere

The contrasts assessing the effect of *Focality* revealed a single significant cluster for the non-focal condition (ONG_Lunf > ONG_Lfoc). The cluster was located in the left fusiform gyrus (*x*, *y*, *z* = 28, − 52, − 16; *T* = 5.73, *p*-FWE-corr. = 0.002), with a symmetrical effect in the right hemisphere (albeit this did not reach full significance after correction for multiple comparisons at the whole-brain level; *x*, *y*, *z* = − 28, − 58, − 10; *T* = 4.83, *p*-FWE-corr. = 0.132). The opposite comparison (focal > non-focal) did not reveal any significant effect.

**Fig. 5:**
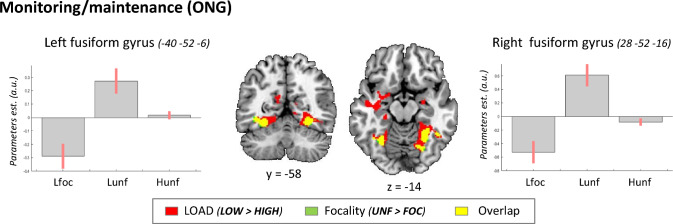
Effects of *Load* and *Focality* during monitoring. Effect of LOW load for non-focal ongoing trials (ONG_Lunf > ONG_Hunf) in red, and effect of UNF task for low-load trials in green (ONG_Lunf > ONG_Lfoc; please note that just a few voxels showed only this effect). Both comparisons activated the fusiform gyrus (overlap rendered in yellow) with activations extending in the parahippocampal gyrus. The signal plots show maximal activity for the condition that combined low-load and non-focal task (ONG_Lunf, central bar in each plot). Activation clusters are significant at *p*-FWE-corr. = 0.05 (cluster level) and are displayed at a voxel-level p-unc. = 0.005, except for the effect of non-focality in the left fusiform gyrus that did not survive correction for multiple comparisons at the whole-brain level (see main text). The signal plots show mean-adjusted parameters estimates ± 95% CI

Figure 5 displays the activation of the fusiform gyrus, where the effects of *Load* (LOW > HIGH) and *Focality* (UNF > FOC) overlapped. The signal plots show the pattern of activation for the three ONG conditions, highlighting that the ventral occipital cortex—extending anteriorly to the parahippocampal gyrus—responded specifically to the low-load non-focal condition (see central bar in both signal plots).

## Discussion

To assess the neural processes involved in prospective memory, we considered conditions where the competition between ongoing and PM tasks arises from multiple sources. We manipulated *Load* and *Focality* of an event-based PM task, expecting that their co-occurrence would impact on the overall executive demands and affect the processing of both ONG and PM stimuli. We hypothesized that, if these two factors draw on common cognitive resources, they should activate overlapping brain regions and possibly interact there. In this respect, our main predictions concerned the dorsal parietal cortex, previously related both to high-load and non-focality, as well as anterior prefrontal regions that former studies implicated in the control of the allocation of resources between internal/intentional and external/sensory sources of information (Burgess et al. 2003; McDaniel et al. 2013). These results only partially supported the initial predictions. In particular, our main finding was that the level of PM *Load* largely determined what brain circuits were involved and whether *Focality* further impacted on brain activity. Specifically, the detection of PM-targets under high-load activated the left IPS in the dorsal parietal cortex, without any further modulation related to *Focality*. By contrast, under low-load, we found significant effects of *Focality* (non-focal > focal) in the ventral occipital cortex both during monitoring and detection. This suggests separate mechanisms underlying the impact of *Load* and *Focality* on the allocation of cognitive resources, as we discussed in the sections below.

### Behavior: the impact of load and focality

Behavioral data confirmed the impact of *Load* on both the monitoring and the detection phases, and that *Load* and *Focality* interacted during detection of the PM1-targets. In the monitoring phase, slower RTs for the high-load condition indicated that participants had to engage cognitive resources to maintain prospective intentions, while at the same time executing the ongoing task (Matos et al. 2020). It is plausible that increasing the number of prospective intentions could have determined a shift from an automatic process to a more consuming monitoring process (Barban et al. 2014). Here, the high-load ONG trials were also non-focal, and this could have further increased the difficulty to process these stimuli, thus requiring additional cognitive resources. Several previous studies that manipulated PM-load interpreted the effect of high-load as a “PM cost” reflecting the interference of PM monitoring on the ONG activity (Einstein et al. 1992; Kidder et al. 1997; Cohen et al. 2008). A novel aspect of the current study is the finding of a “PM cost” that was also present in the detection phase. Specifically, we found that the increase in the number of intentions to be executed (PM1 plus PM2-targets in the high-load condition) affected the detection of the primary PM1-targets.

The behavioral results revealed an interaction between *Load* and *Focality* on the PM1 reaction times. However, contrary to our initial expectations, the slowest performance was found for the focal high-load trials, rather than for the non-focal high-load trials. The RTs also showed that in the high-load condition, participants were faster to respond to the PM2-targets than the PM1-target (656 vs. 683 ms; t(14) = 3.13, *p* = 0.007). This suggests that participants gave higher priority to the PM2-targets than to the PM1-targets, which may explain the unexpected patterns related to the PM1 focality. Specifically, PM1 and PM2-targets always required judging different dimensions (color vs. letter). Thus, in the high-load condition, when the PM1-target was focal (i.e. same dimension as ONG), these PM1-targets were actually "non-focal" with respect to the high priority PM2. Accordingly, we suggest that here the interaction between *Load* and *Focality* during the detection of high-load PM1-targets entailed the requirement of overcoming potential interference related to the PM2 intention. Future studies should investigate this more systematically, for example using 3 different dimensions (e.g. letter-color, letter-identity, letter-case) and test conditions that would involve judging different combinations of dimensions between the ONG/PM1/PM2 tasks. This would enable varying the focality of the three tasks in an independent manner, and to directly test how the processing priority given to PM1 vs. PM2 affects the competition between the three tasks.

### fMRI: monitoring and detection networks

The direct comparison between ONG and PM trials revealed activation of the anterior PFC (BA 10, BA 11) and the posterior cingulate cortex (PCC); see Fig. 3a. The engagement of these regions in consistent with previous imaging work that associated the anterior PFC with the maintenance of prospective intentions (e.g. Burgess et al. 2001; 2003; den Ouden et al. 2005; Gilbert et al. 2009; Momennejad and Haynes 2012; Okuda et al. 1998, 2011; Burgess et al. 2011) and PCC with monitoring operations (Reynolds et al. 2009; Simons et al. 2006; Gilbert et al. 2009).

The reverse comparison (PM > ONG) revealed activation of a wide range of areas, comprising dorsal frontoparietal regions (including the IPS), occipital cortex, insula, SMA, and the putamen. Based on the *A-to-DI* model (Cona et al. 2015), we expected that the detection phase would activate the ventral fronto-parietal network, and in particular the inferior parietal lobule. By contrast, our results highlighted extensive activation in the dorsal fronto-parietal cortex (see Fig. 3b). We suggest that the prominence of dorsal activity here reflects the high cognitive demands of the current task that included targets and distractor stimuli displayed on each trial, plus the performance of 2 or 3 concurrent tasks (ONG, PM1, plus PM2 in the high-load condition, see also main effect of high-load in left IPS, Fig. 4a and discussion below). The high demand of cognitive resources is likely to require strategic processes previously associated with the dorsal frontoparietal network (Cona et al. 2015, 2017; Burgess et al. 2011). Moreover, it should be noticed that our main analyses compared only correct trials. Dropping this constraint for the "PM > ONG contrast" revealed additional activation of the inferior parietal cortex. This highlights the central role of dorsal areas for the successful control of task-based resource allocation, while ventral regions may engage in a more automatic manner during detection, here sometimes yielding to erroneous responses.

### Detection and monitoring: the impact of load

Using a targeted ROI analysis of the left IPS, we found that the detection of high-load PM-targets (i.e. PM2 and high-load PM1) activated this region regardless of task focality. The IPS is a central node of the dorsal attention system that mediates the allocation of top–down attention to the environment (Corbetta and Shulman 2002) and has also been associated with voluntary memory retrieval (see Ciaramelli et al. 2008). As a part of the dorsal frontoparietal network, the IPS is responsible both for maintaining intentions and for active monitoring of PM-targets, thus mainly mediating top–down strategic PM processes (McDaniel et al. 2013; Cona et al. 2015). Here, we focused on the left IPS based on previous evidence of left-hemisphere lateralization during the detection of high-load PM-targets (Cona et al. 2017). Our current finding of left IPS activation during the detection of high-load PM-targets fits with the view that this region combines attentional/perceptual and memory-related goals for top–down control (see also Seibert et al. 2011).

Further, our results emphasize the relevance of *Load*, over *Focality*, for the activation of the dorsal attention system. The previous literature already pointed out that only some PM tasks require the frontoparietal attention network to efficaciously maintain delayed intentions (McDaniel et al. 2015). Our current results in the detection phase extend this and suggest that the increasing demands associated with the monitoring of two PM-targets (PM1 and PM2) also impact on the detection processes. By contrast, *Focality* did not affect the dorsal parietal cortex. While we do not wish to overemphasize this "negative" finding as regard to focality, this pattern suggests that—contrarily to our initial expectations (cf. Introduction)—*Load* and *Focality* do not rely on shared cognitive resources.

In the detection phase, we also directly compared high-load PM1 trials versus PM2 trials. While these two trial types entail the same level of load (i.e. high), they were associated with different behavioral performances (faster detection of PM2 than PM1-targets, see above). The imaging analyses revealed greater activation in the precuneus, parahippocampus, right hippocampus, and the right insula for PM1 compared with PM2-targets. Activation of the precuneus has been frequently reported in previous studies of PM (den Ouden et al. 2005; Eschen et al. 2007; Gilbert et al. 2009; Hashimoto et al. 2011; Okuda et al. 1998, 2007, 2011; Poppenk et al. 2010; Reynolds et al. 2009; Simons et al. 2006), also highlighting its role in the detection of PM-targets in high-load conditions (Barban et al. 2014). The precuneus is part of a posterior circuits linking long-term memory processes in the hippocampus with visuo-spatial online processing in the posterior parietal cortex (Byrne et al. 2007). Here, we suggest that the activation of the precuneus and the medial temporal cortex for the high-load PM1-targets reflects the retrieval of "low-priority" intentions, as opposed to "high priority" PM2 (cf. behavioral data). Notably, at a brain imaging level, the behavioral cost of PM1 vs. PM2 detection dissociated from the overall effect of high-load that instead activated the left IPS, cf. above. Thus, different factors involving an increase of cognitive effort (as measured using RTs) lead to the activation of different sets of brain regions, highlighting that PM entails multiple process-specific resources, as opposed to common/shared resources; see also previous paragraph.

In the monitoring phase, we found that ONG trials at low levels of load activated the ventral occipital–temporal cortex plus the insula bilaterally and the cingulate cortex. Considering the role of the ventral occipital cortex in the processing of form, color, and visual features (Ungerleider and Mishkin 1982), its contribution here may reflect the allocation of processing resources to identify letters/colors, while monitoring a single PM-target in the low-load condition. We suggest that the insula and the posterior cingulate cortex contribute to this process by allocating resources to the external environment, disengaging them from internal monitoring (Hampson et al. 2006; Gilbert et al. 2007; Pearson et al. 2011; Cona et al. 2015) and allowing cognitive control to operate according to early selection mechanisms. By contrast, such early selection mechanism would not be at play in the high-load conditions, when the strategic allocations of resources—and internally directed processes—are needed to handle the competition between the ONG task and two concurrent PM tasks (see also below, findings that focality modulated activity in the same ventral occipital–temporal areas only in the low-load conditions).

### Monitoring and detection: the impact of focality

A main prediction of the study was that, if *Focality* and *Load* rely on shared cognitive control, the non-focal conditions would activate the same regions as the high-load conditions, possibly including dorsal parietal cortex and/or the rostral prefrontal cortex (see “Introduction”). This prediction was not satisfied neither during PM detection nor for the ONG monitoring phase.

During detection of PM1-targets, neither a targeted ROI analysis of the left IPS nor the whole-brain analysis revealed significant interactions between *Focality* and *Load*. At a low, uncorrected threshold, we found a cluster of activation in the rostral part of the middle frontal gyrus (*x*, *y*, *z* = 30 46 2, *T* = 3.78, *p*-unc. < 0.001). Contrary to our expectations, activity in this region was highest for the non-focal trials in the low (rather than high) load condition. Nonetheless, we briefly discuss this activation here, given that many previous studies reported prefrontal activation in PM tasks. In the light of the *Gateway Hypothesis* (Burgess et al. 2007), the prefrontal cortex has been associated with the control of resources between internal/intentional and external/sensory sources of information (Burgess et al. 2003; McDaniel et al. 2013; Barban et al. 2014). Further, Barban and colleagues (2020) showed that the lateral and the medial portions of the aPFC are involved in voluntary monitoring vs. spontaneous reflexive-associative processes, respectively. Both regions would be recruited during PM tasks and the predominance of one region over the other would depend on several factors, including the focality of the task. The cluster observed in the current study was located relatively laterally, and the further modulation according to *Load* may be consistent with the role of this region at the interface between prospective memory and (voluntary) attention control.

By contrast, in the ONG monitoring phase, we found robust effects of *Focality*. The non-focal ONG trials activated the ventral occipital cortex, showing considerable overlap with the effect of *Load* (see Fig. 5). Specifically, the ventral occipital cortex activated mostly for the non-focal, low-load trails. As noted above, we interpreted this as an effect of an early selection mechanism that would be at play specifically in the low-load condition, when there are cognitive resources available for externally oriented processes. This would be in line with the notion of "constant target-checking" that constitutes a signature of the monitoring phase (Gonneaud et al. 2014). Sensory modulation would contribute to target-checking by selecting the relevant stimulus dimension within the visual cortex, when—in low-load non-focal trials—participants had to pay attention to one dimension for the ONG trails and a single, but different, dimension for the PM1-targets (see also Kalpouzos et al. 2010). Thus, we suggest that in the low-load non-focal ONG trials, cognitive control operated via early selection mechanisms by prioritizing the allocation of resources to target-checking via modulation of sensory processing.

A main limitation of the current study was the relatively small sample size, which reduced the statistical power of our analyses. We recruited a total of 18 participants, and due to data quality issues, we had to exclude 3 participants, resulting in a final sample size of 15 participants. This sample size may have limited our ability to detect a significant interaction between Load and Focality during the detection of the PM1-targets. Underpowered designs may also lead to false-positives due to poor sampling of the true underlying population effect, yielding to the reporting of large effects even when the true effect is small or absent (Szucs and Ioannidis 2020; but see also Lieberman and Cunningham 2009). Nonetheless, the current results provide us with new insights into the complex interplay between *Load* and *Focality* and can serve as the basis for future studies that, including larger sample sizes, could assess how the task-features (focality) of ONG, PM1, and PM2 impact on the allocation of processing priorities under high-load conditions (see discussion of the behavioral results, above).

## Conclusion

The present study sought to shed light on the contribution of *Load* and *Focality* in accomplishing a PM task, separately addressing the monitoring and detection phases. Our findings point to the evidence of distinct mechanisms subtending complex everyday PM behavior (*multiprocess framework*). Results suggest that *Load* and *Focality* operate via different mechanisms, with level of task *Load* largely determining how cognitive control operates to select relevant information, prioritizing the allocation of cognitive resources to internal/intentional or external/sensory sources. At high levels of load, the detection of PM-targets depends on internal/strategic processes instantiated via the dorsal attention network (here IPS), and task focality plays a lessened role on the target selection process. By contrast, at low levels of load, more resources are available and these are allocated to modulate processing in sensory areas (here, the ventral occipital cortex). In turns, this discloses different selection requirements according to task focality, with non-focal tasks involving more competition between ONG and PM and greater activity in ventral occipital cortex both during monitoring and detection. To conclude, the current findings provide us with some initial insight into the complex effects that arise when multiple factors jointly contribute to prospective intentions, and highlight that this does not merely entail the engagement of a centralized system controlling some shared executive resources.

## Data Availability

The dataset generated and analyzed during the current study is available from the corresponding author on reasonable request.

## Abbreviations

ONG: Ongoing task
PM: Prospective memory
IPS: Intraparietal sulcus
MFG: Medial frontal gyrus
aPFC: Anterior prefrontal cortex
SMA: Supplementary motor area
vmPFC: Ventro-medial prefrontal cortex
